# What is meant by “frailty” in undergraduate medical education? A national survey of UK medical schools

**DOI:** 10.1007/s41999-021-00465-9

**Published:** 2021-03-02

**Authors:** Rebecca Winter, Muna Al-Jawad, Juliet Wright, Duncan Shrewsbury, Harm Van Marwijk, Helen Johnson, Tom Levett

**Affiliations:** 1 Department of Medical Education, Brighton and Sussex Medical School, Room 344A Mayfield House, Falmer, Brighton, BN1 9PH UK; 2 Department of Primary Care and Public Health, Brighton and Sussex Medical School, Brighton, BN1 9PH UK; 3 School of Applied Social Science, University of Brighton, Brighton, BN1 9PH UK; 4 Department of Clinical and Experimental Medicine Brighton, Brighton, BN1 9PH UK

**Keywords:** Frailty, Teaching, Assessment, Medical education, Undergraduate, Curriculum

## Abstract

**Purpose:**

All UK medical schools are required to include frailty in their curriculum. The term is open to interpretation and associated with negative perceptions. Understanding and recognising frailty is a prerequisite for consideration of frailty in the treatment decision-making process across clinical specialities. The aim of this survey was to describe how frailty has been interpreted and approached in UK undergraduate medical education and provide examples of educational strategies employed.

**Methods:**

All UK medical schools were invited to complete an electronic survey. Schools described educational strategies used to teach and assess frailty and provided frailty-related learning outcomes. Learning Outcomes were grouped into categories and mapped to the domains of Outcomes for Graduates (knowledge, skills and values).

**Results:**

25/34 Medical schools (74%) participated. The interpretation of what frailty is vary widely and the diversity of teaching strategies reflect this. The most common Learning outcomes included as “Frailty” are about the concept of frailty, Comprehensive Geriatric Assessments and Roles of the MDT. Frailty teaching is predominantly opportunistic and occurred within geriatric medicine rotations in all medical schools. Assessments focus on frailty syndromes such as falls and delirium.

**Conclusion:**

There is variation regarding how frailty has been interpreted and approached by medical schools. Frailty is represented in an array of teaching and assessment methods, with a lack of constructive alignment to related learning outcomes. Consensus should be agreed as to what frailty means in medical education. Further research is required to explore which frailty-specific educational strategies in undergraduate medical education enhance learning.

## Key summary points

**Aim:**

UK medical schools are required to teach about frailty but the term is open to interpretation. This national survey aims to understand how frailty has been perceived and approached by schools.

**Findings:**

Frailty is perceived and approached in a broad variety of ways ranging from a long term condition to geriatric medicine in its entirety. A range of educational approaches have been used to teach and assess, with little constructive alignment to match learning outcomes. Teaching is most commonly opportunistic, by a student observing geriatric ward rounds.

**Message:**

Frailty is open to individual interpretation. Expert consensus should be reached regarding the core areas to include in UGME around the topic of frailty. It would be prudent to explore which frailty-related educational strategies enhance student knowledge, attitudes and values towards frailty.

## Introduction

People living with frailty account for 20% of hospital inpatients and half of all hospital bed days in the UK [1]. Medical students will encounter many patients with frailty, across a breadth of clinical specialities and clinical conditions, which requires an in-depth understanding of frailty. The understanding and recognition of frailty is a prerequisite for consideration of frailty in the treatment decision-making process [2]. The 2018 General Medical Council (GMC) Outcomes for Graduates (OfG) document reflects this, with a requirement that medical schools include frailty in their curriculum [3].

The challenges associated with teaching and learning about frailty include negative perceptions, lack of a universal definition and variation in the interpretation among healthcare professionals [4–7]. There is minimal evidence around medical students’ perception and understanding of frailty. Nimmons et al. found that medical students do not recognise the concept of frailty [8], whilst Mccarthy et al. found that students consider ageing and frailty differently, with frailty attracting more negative associations [9]. A systematic review in postgraduate education found no relevant publications addressed the evidence of educational programmes for frailty prevention and/or frailty management [10]. This gap in training of healthcare professionals has been highlighted as a main barrier towards identifying and managing frailty [11]. There is some evidence to suggest that with teaching the perceived importance and competence in assessing, diagnosing and managing frailty can improve [12].

The aim of this national survey is to outline the interpretation and approach to frailty in Undergraduate Medical Education (UGME) in UK medical schools, to provide a cross section of current practice and describe planned changes to meet the OfG recommendations [3].

## Method

The Brighton and Sussex Medical School (BSMS) Research Governance and Ethics Committee (RGEC) granted ethical approval to proceed with this project in December 2018. (Reference: ER/BSMS9638/1).

The survey was generated using Qualtrics software [13], an electronic survey tool. Questions were designed by consensus, based on the British Geriatric Society (BGS) Good Practice Guide for Frailty [14], national frailty guidance [15] and similar national surveys [16–19]. The survey was piloted by senior members of faculty at one UK medical school, adapted following feedback and reviewed and endorsed for circulation by the BGS Education and Training Committee. The survey included open and closed questions, with free text boxes to expand on responses. Schools were asked to provide frailty-related learning outcomes (LOs). All respondents were invited for a follow up telephone call to clarify and expand on responses.

The medical education leads (MELs) from 34 UK medical schools were contacted through the Medical School Council. The MELs nominated a representative with knowledge of frailty in their undergraduate medical curriculum. Three routine reminders were sent. Non-responding schools were then contacted once by education representatives from the BGS Trainees Council.

Data were collected over 6 months. Medical schools were anonymised and randomly assigned a code between M1 and M25. Responses from the survey and telephone follow up were analysed using descriptive statistics and descriptive content analysis; a systematic approach used for exploring large amounts of textual information to describe the characteristics of the document’s content [20]. To demonstrate the variety of content included under the term frailty, the frailty-related LOs were grouped into categories by three authors (RW, MAJ, TL). These authors collectively mapped each LO to a domain within OfG (knowledge, skills or values).

## Results

Responses were received from 74% (25/34) of medical schools. 56% of respondents (14/25) participated in follow up telephone calls. 92% (23/25) of respondents held both clinical and academic roles. Of these, 70% (16/23) were the lead of geriatric medicine (GM) modules and 87% (20/23) were consultant geriatricians.

### Teaching about frailty

Frailty is taught in 80% (20/25) of responding schools. Where frailty is taught, 100% (20/20) indicated that frailty teaching occurred within GM modules; exclusively so in 50%. Geriatricians contribute to frailty teaching in all cases alongside other professionals. However, in 20% of cases, only geriatricians deliver frailty teaching. 80% of schools described combinations of multidisciplinary team (MDT) members as faculty, with a minimum of three disciplines (for example geriatrician, occupational therapist, physiotherapist). In 25% of schools, General Practitioners deliver teaching on frailty.

The content of frailty teaching includes: the definition of frailty (100%); frailty screening and assessment tools (95%); roles of the MDT in frailty care (95%); frailty diagnosis (90%); and frailty management (90%). 55% of schools taught about frailty prevention. 25% described that students learn about frailty through completing or observing the Clinical Frailty Scale (CFS): “Students encounter frailty in the form of the clinical frailty scale being done on all patients when they come into hospital”. *(M7).*

Most medical schools (95%) perceive that frailty teaching occurs opportunistically on clinical placement: “Students should get some exposure to clinical geriatrics during medical ward attachments and this will include patients with frailty” *(M25) and* “Frailty [is] not formally, specifically delivered to all however likely to crop up…” *(M7).* Of formal teaching, 85% (17/20) teach about frailty via case studies, typically based around patients presenting with a fall, delirium or a chronic condition such as heart failure. 80% use small group teaching and 80% use lectures. 45% teach via community visits. Only 30% of medical schools use computer aided learning to teach about frailty. Four examples of teaching are summarised in Table 1, reflecting the diversity of approaches.

**Table 1:** Examples of teaching in practice

Modality	Content
Pre-reading of two seminal papers on frailty and sarcopenia [41, 42] Completion of a case-based workbook Online learning module Small group discussion Clinical skill sessions	Based around a patient with heart failure who has frailty. Students need to complete a workbook and decide the patient’s frailty status based on the phenotype and cumulative deficit models. CGA is taught by a variety of modalities and faculty and clinical skills sessions on gait and balance assessment is delivered by physiotherapists. *(M23)*
A frailty teaching ward round (WR)	Led by a Teaching Fellow (TF), within and parallel to the consultant WR. The TF identifies and highlights key learning opportunities to the students. Students are taken aside for a short tutorial led by the TF, running parallel to the WR, to discuss a topic in more depth, to help break down the complexity and uncertainty surrounding frail patients. *(M3)*
Online tutorial and video Small group discussion	Introduce normal ageing and then frailty, including watching ‘Mrs Andrews’ story’ [43]. Important learning points are discussed in small groups around a clinical case. *(M11)*
Year-long longitudinal primary care placement Reflective writing	Students attend placement weekly with a specific focus on patients with frailty. They complete a CGA and management plan on a patient they have seen Students undertake a three-part reflective written piece on the meaning of frailty from the perspectives of the patient, MDT and student, bringing in a critical evaluation of their management plan and the literature around frailty*. (M10)*

In 65% of cases, frailty is taught in 2 year groups, most commonly years 2 and 4 although several combinations of a junior year (years 1, 2) and senior years (3,4,5,6) were provided. Year 4 receives frailty teaching in 65% of medical schools and were the year group most commonly assessed on frailty (75%). 20% of medical schools teach frailty with students from other professions. These include prescribing workshops in two medical schools, with medical students, pharmacy students and nursing students. Other interprofessional education (IPE) sessions included teaching around ethical issues in ageing and frailty, and a seminar about discharge planning.

### Learning outcomes

60% (12/20) Of schools provided LOs relevant to frailty, demonstrating a variation in how frailty has been interpreted by schools. Sixty-one learning outcomes were received and grouped into fourteen categories (Table 2). LOs were mapped to the domains in the OfG document; thirteen LOs covered more than one domain. Knowledge was represented most commonly (40/61); followed by values (18/61) and skills (16/61). Only 17% (2/12) of medical schools provided LOs spanning all three domains.

**Table 2:** Frailty learning outcomes and their categories

Category	Example learning outcome	Proportion of LOs in each category
The concept of frailty	Understand and describe the concept of frailty *(M4)*	8/61
CGA	Understand what comprehensive geriatric assessment is, its importance; be aware of the evidence supporting this approach in improving health outcomes in frail older people. *(M17)*	8/61
MDT roles, and discharge planning	Recognition of the need to work constructively and considerately with other members of the MDT, family and carers *(M3)*	7/61
Impact of illness on the older adult	Recognise that ill health in older people is often due to a complex mix of medical, physical, psychological, and social problems. *(M15)*	6/61
Social impact of ageing	Consider the burden imposed on the healthcare system by an increasing aging population and what strategies might be employed to manage this *(M10)*	4/61
Polypharmacy in an older adult	Recognise drug actions, therapeutics, pharmacokinetics, drug side effects, the effects of polypharmacy, the principles of safe drug prescription in the older person *(M11)*	4/61
Chronic conditions in the older person	Understand the basic principles and options / strategies for “Chronic Disease Management” in the Older Patient. For e.g. CCF; COPD; Dementia; Chronic Leg Ulcers with reduced Mobility; Osteoporotic fractures; Cerebrovascular Disease; Parkinson’s *(M2)*	4/61
Ethical and legal topics around the older patient	Assess patient’s capacity in accordance with legal requirements and GMC guidance. This includes the Mental Capacity Act 2005, Deprivation of Liberties Safeguards (DoLS) and the role of Best Interest Decisions *(M11)*	3/61
ACP, EOL Care and Death	Understand the basic principles of “Realistic Medicine,” “Advance Care Planning,” “Anticipatory Care Planning,” “End of Life Care” / Palliative Care (especially in relation to Older Patients) *(M2)*	3/61
Frailty scores	Describe some common frailty scores. *(M1)*	3/61
Frailty syndromes	To describe frailty syndromes in context of Isaac’s Geriatric Giants *(M25)*	3/61
Investigations	Initiate, justify and interpret appropriate haematological, biochemical, radiological and other relevant investigations pertaining to the clinical condition(s) *(M11)*	3/61
Multimorbidity and disability	Assess the functional ability of a patient, relating function and disability to the underlying disease process *(M10)*	3/61
Gerontology	To develop an understanding of the biology of Ageing and the factors that can promote/ lead to healthy ageing *(M4)*	2/61

### Assessments on frailty

Most schools (90%, 18/20) assess frailty. One school reported teaching without assessments about frailty and another assesses frailty without teaching.

A variety of formal assessment methods relating to frailty were described. Objective Structured Clinical Exams (OSCE’s) (85%) and Single Best Answer (SBA) (70%) were the most common formative methods used. Examples provided include questions around older patients presenting with frailty syndromes (namely falls and delirium) rather than specific questions about frailty. Case discussion (45%), reflective writing (30%) and Short Answer Questions (25%) were also used. Summative assessments including logbook completion and Workplace Based Assessments were common.

### Planned GMC changes

60% of medical schools (15/25) have planned changes to meet the OfG recommendations surrounding frailty. One medical school plans to evolve their programme to include more frailty based on the BGS undergraduate curricula; [21] three schools are extending the time allocated in primary care placements to deliver frailty teaching in the community, although it is unclear what this means in practice. All schools that do not teach or assess frailty are planning curriculum changes.

## Discussion

The survey provides the first analysis of teaching and assessment of frailty in UGME; exploring how frailty has been interpreted and approached by UK medical schools through descriptions, examples and LOs. The majority of schools identified that frailty is being taught and assessed in their institution yet there is significant variation in perception of what frailty is and how frailty teaching has been interpreted by medical schools. Frailty is taught using an array of educational approaches ranging from opportunistic teaching on ward rounds to small group learning to reflective writing. There appeared to be little consensus as to which aspects about frailty should be considered core in UGME but within the LOs provided, the concept of frailty, CGA and the roles of the MDT featured most commonly.

Some of the provided LOs represented frailty as a long-term condition within GM (the concept of frailty, frailty assessment tools such as the Clinical Frailty Scale) and others considered frailty to equate to the whole of GM (chronic conditions in the older patient, social impact of ageing, gerontology). This reflects ongoing discussion amongst academics and clinical educators [22], yet in terms of education frailty is currently mentioned within both the British and European Undergraduate Curriculum in GM as opposed to representing the GM curriculum in its entirety: “Discuss the generalisability of existing research studies to frail older people…” [21] and “Define the concept of frailty in older people” [23]. A curricular update including the position of these influential bodies of how frailty is best situated in UGME is required.

Of the LOs provided, knowledge about frailty was the OfG domain represented most commonly. Only two schools provided LOs spanning all three OfG domains. OfG now discusses the domain of values, not attitudes but our findings mirror previous surveys describing UK undergraduate teaching on delirium and dementia, which also identified failure to address student attitudes [18, 19]. Whilst medical students’ attitudes towards older patients are acknowledged [24, 25], little is known regarding medical students’ attitudes towards patients with frailty. A qualitative study found that fourth year medical students did not recognise frailty as a medical entity or demonstrate an understanding of management principles [8]. Another study of UK medical students found that students consider ageing and frailty as different concepts, with frailty attracting more negative associations [9].

Frailty teaching was widely delivered through ward rounds and clinical placements, commonly opportunistically. Simply being present in a clinical environment, however, does not guarantee that students will recognise or understand the concept of frailty. Furthermore, the nature of frailty means that people often present in an atypical manner, with multiple comorbidities and changes in functional ability, which can be challenging even for experts. Many schools use a systems-based approach to learning. Given that frailty is a multi-system condition, it is unclear of the optimal stage in undergraduate training to introduce teaching and assessment about frailty.

A number of schools discussed that students learn about frailty by completing or observing the CFS [26]. The CFS was designed to enable frailty to be measured in the outpatient clinical setting, not to teach about frailty [15]. It is often completed by the most junior member of the team, when a patient is acutely unwell. As an opportunistic teaching tool, the visual scale may over-simplify frailty when trying to teach the complexity and nuance of frailty.

Patients are currently included in frailty teaching opportunistically in the clinical environment. This is a missed opportunity as it is recognised that patient-educators have a significant role in supporting students’ learning. Their personal insights could be crucial as there are known negative perceptions towards frailty among older persons [5, 27].

There is a wealth of data regarding the relevance of frailty on patient outcome in clinical specialties other than geriatric medicine [28–31]. However, this survey suggests that currently geriatricians hold the responsibility for delivering frailty teaching in UGME. It also suggests that the gatekeeper, in this case a senior member of medical school faculty, perceives that frailty is the responsibility of GM. The extent of this positioning on what students learn about frailty is unclear. The concern is that restricting frailty to GM rotations may be too reductive as students must be aware that patients they encounter on non-geriatric placements have frailty, and appreciate that the same principles of presentation and management apply.

Only a small proportion of medical schools currently teach about frailty in the community. It is compulsory for foundation doctors to undertake a community placement and is a key recommendation for medical students in OfG [3, 32, 33]. Furthermore, there is an increasingly recognised role of primary care in identifying and managing frailty; in 2017 NHS England introduced a new requirement for all general practices to identify and appropriately manage all patients over the age of sixty-five with moderate or severe frailty [34, 35].The most commonly reported medical school change to meet OfG included increasing time that students spend in primary care.

A multi-disciplinary approach is required to meet the complex care needs of older people living with frailty [15]. 80% of medical schools use at least 3 members of the MDT to teach about frailty. Conversely, the proportion of schools framing interprofessional education (IPE) around frailty was low at 20%. IPE occurs when students from two or more professions learn about, from and with each other to improve health outcomes. It has been shown to be effective in positively changing patient outcomes in the topics of delirium, dementia and falls [36–38] and is advocated in OfG [3].

The assessment examples and descriptions most commonly described OSCE scenarios, including a history or communication station involving a frailty syndrome (fall, episode of delirium) or prescribing stations demonstrating polypharmacy. The examples do not appear to reflect the LOs provided, nor the diversity of the teaching that has been described. For example, all medical schools teach the definition and diagnosis of frailty, yet none assess this. This reflects a lack of constructive alignment; where all components in the educational system including the LOs, teaching methods and assessment methods should align to each other [39]. Assessment has been shown to drive learning and there is a significant link between the weighting of a subject within an assessment scheme and medical students’ reported motivation towards learning the subject [40]. The Medical Licencing Assessment (MLA) is a national examination, based on the blueprint of OfG and will commence in 2023 [3]. Consensus should be reached prior to the commencement of the exam of how frailty will be best assessed.

A strength of this study is the high response rate for a survey of this type, capturing a breadth of experiences, with participation maximised through a number of recruitment measures. The survey has some limitations. Not all UK medical schools were represented and data were collected from one individual at each medical school. This may introduce bias due to their knowledge of their curriculum. It can be challenging to assess individual performance; a person with greater knowledge about frailty may paradoxically be more likely to evaluate themselves poorly. In this survey two schools that identified they did not teach about frailty showed an insightful understanding of frailty. Lastly, the integrated nature of frailty across multiple conditions and body systems alongside the nature of some medical schools’ curricula meant that the structure of a survey may have made it more difficult for schools to detail responses and extract frailty-related LOs. However, to align with the OfG document, medical schools will need to be able to map frailty to their curriculum.

We acknowledge this survey focusses on the UK picture only. The literature surrounding frailty-specific teaching and learning is currently restricted to the UK and Australia [8, 9, 12]. Owing to the presence of frailty-related learning outcomes in the European Medical Undergraduate Curriculum [23], replicating the survey within Europe would provide a broader picture and share areas of good practice.

We recommend that expert consensus should be reached regarding the core areas to include in UGME around the topic of frailty. This should be included in future versions of the undergraduate curriculum produced by learned societies such as the BGS and the European Geriatric Society. In view of the variation in frailty-related educational strategies, it would be prudent to explore which strategies enhance student learning around frailty. Medical schools should ensure that frailty-related LOs are taught and assessed to follow the principles of constructive alignment. Furthermore, there should be careful consideration as to how frailty assessments feature within the national MLA. Further research is required to understand how medical students and clinical teachers across specialties perceive frailty, how these perceptions impact what students learn in the clinical environment, and how frailty being positioned within geriatric medicine impacts on student learning.

In summary, frailty is complex, open to interpretation and negatively perceived. This is the first survey to explore frailty in UGME and describes the how frailty has been interpreted and approached in diverse ways in UK curricula. Recommendations are provided to lead the way in preparing tomorrows doctors for the patients of the future.

## Data Availability

Available on request to main author.
